# Impact of the COVID-19 pandemic on body mass index in children and adolescents after kidney transplantation

**DOI:** 10.1007/s00467-023-05902-4

**Published:** 2023-03-02

**Authors:** Nele Kirsten Kanzelmeyer, Friederike Weigel, Johannes Boeckenhauer, Dieter Haffner, Jun Oh, Raphael Schild

**Affiliations:** 1 Department of Pediatric Kidney, Liver and Metabolic Diseases, Hannover Medical School, Carl-Neuberg Str. 1, 30625 Hannover, Germany; 2 Division of Pediatric Nephrology, University Children’s Hospital, Jena, Germany; 3 Division of Pediatric Nephrology, University Medical Center Hamburg-Eppendorf, Hamburg, Germany

**Keywords:** COVID-19, Kidney transplantation, Weight, Children, Adolescents, Blood pressure

## Abstract

**Background:**

The coronavirus SARS-CoV-2 disease (COVID-19) pandemic affected lifestyles and resulted in significant weight gain in the general population. Its impact on children after kidney transplantation (KTx) is unknown.

**Methods:**

We retrospectively evaluated body mass index (BMI) *z*-scores during the COVID-19 pandemic in 132 pediatric KTx patients, followed-up at three German hospitals. Among those, serial blood pressure measurements were available for 104 patients. Lipid measurements were available from 74 patients. Patients were categorized according to gender and age group, i.e., children versus adolescents. Data were analyzed by a linear mixed model approach.

**Results:**

Before the COVID-19 pandemic, female adolescents presented with higher mean BMI *z*-scores compared to male adolescents (difference: − 1.05, 95% CI − 1.86 to − 0.24, *p* = 0.004). No other significant differences could be observed among the other groups. During the COVID-19 pandemic, the mean BMI *z*-score increased in adolescents (difference: male, 0.23, 95% CI 0.18 to 0.28; female 0.21, 95% CI 0.14 to 0.29, each *p* < 0.001), but not in children. The BMI *z*-score was associated with adolescent age, and with the combination of adolescent age, female gender, and the duration of the pandemic (each *p* < 0.05). During the COVID-19 pandemic, the mean systolic blood pressure *z*-score significantly increased in female adolescents (difference: 0.47, 95% CI 0.46 to 0.49).

**Conclusions:**

During the COVID-19 pandemic, adolescents in particular showed a significant increase in their BMI *z*-score after KTx. Additionally, an increase in systolic blood pressure was associated with female adolescents. The findings suggest additional cardiovascular risks in this cohort.

**Graphical abstract:**

**Figure:**
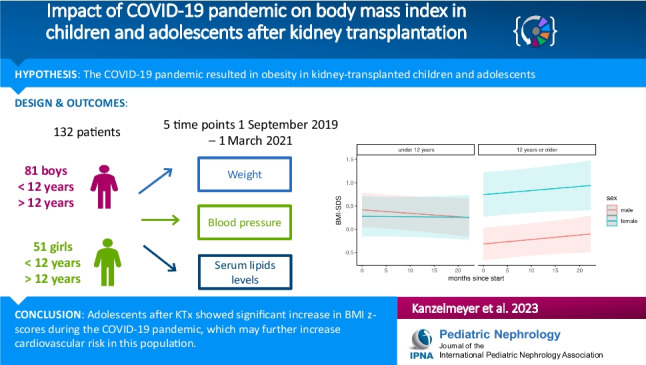
A higher resolution version of the Graphical abstract is available as Supplementary information

**Supplementary Information:**

The online version contains supplementary material available at 10.1007/s00467-023-05902-4.

## Introduction

The coronavirus SARS-CoV-2-disease (COVID-19) pandemic led to lifestyle changes, restrictions on social relationships, and curtailed activities in the general population. The associated lockdowns caused modifications in diet and physical activity, and increased psychological distress [1]. Those changes affected not only adults but also toddlers, school-age children, and particularly adolescents [2].

Obesity is a rising public health threat in both pediatric and adult populations, and its prevalence in the healthy general population has significantly increased over the past five decades [3]. This alarming trend in obesity is multifactorial. Massive weight gain increases the risk of developing comorbidities such as diabetes mellitus, metabolic syndrome, hypertension, and cardiovascular disease [4]. Consequently, the increase in obesity prevalence leads to an increase in those associated comorbidities, resulting in an enormous burden of obesity-related diseases worldwide. The obesity risk in children is especially high after a solid organ transplantation. Cross-sectional studies of pediatric transplant recipients revealed that obesity rates range from 10 to 30%, were particularly present in teenage years, and were associated with poorer long-term graft survival and function [5–7]. Due to concomitant immunosuppressive medication, these patients have an increased risk of infection-associated hospitalization and often show reduced physical activity. Potentially, this could have further worsened during the COVID-19 pandemic. But despite the COVID-19 pandemic being linked to significant weight gain in the general pediatric population [8], its impact on high-risk populations like pediatric kidney transplant recipients is largely unknown. Therefore, we retrospectively assessed the changes in BMI *z*-scores and their determinants in a cohort of children and adolescents after kidney transplantation (KTx) during the COVID-19 pandemic, followed-up at three German pediatric nephrology centers.

## Research design and methodology

### Population and study design

This retrospective study included pediatric KTx recipients (age < 18 years) with a stable graft function followed up between September 1, 2019 and September 1, 2021 at the pediatric nephrology centers of Jena, Hamburg, and Hannover, Germany. Patients were assessed five times over a period of 2 years, i.e., 6 months before the start of the COVID-19 pandemic in Germany (September 1, 2019), at the start of the COVID-19 pandemic in Germany (March 1, 2020) and thereafter in intervals of 6 months (September 1, 2020, March 1, 2021, and September 1, 2021). Age, gender, weight, and height were assessed. A total of 132 patients (51 girls and 81 boys) were eligible for analysis for whom data were available in September 2019 and at least one subsequent time point after March 1, 2020. There was no loss of follow-up concerning weight, height, and BMI. All patients underwent KTx before the study entry. The study sample did not include any patients with combined transplantations. Blood pressure, total serum cholesterol, low-density cholesterol, and high-density cholesterol were evaluated as additional parameters in a subcohort.

### Measures

For each time point, BMI was calculated as weight in kg/(length in meters)^2^. Thereafter, age-dependent *z*-scores were calculated using national reference values [9]. Patients were classified according to the German guideline on obesity in children [10] as underweight (BMI *z*-score <  − 1.28), normal (BMI *z*-score =  − 1.28 to 1.28), overweight (BMI *z*-score = 1.29 to 1.88), or obese (BMI *z*-score > 1.88), respectively. Patients were categorized according to chronological age as children (age < 12 years) or adolescents (age 12 < 18 years), and gender (female or male). For the subgroup analysis, relevant positive and negative changes in the BMI *z*-score were defined as ≥ 0.2/year and ≤  − 0.2/year, respectively. Blood pressure values and serum lipid levels were compared with reference values obtained from healthy children [11, 12].

### Statistical analysis

We described continuous variables as mean (SD) and categorical variables as counts (percentage). To model the time trend of the BMI *z*-score, we applied a linear mixed-effects model using the lme4 package of R [13, 14]. Besides the fixed effects of age group, sex, time, and their respective interactions, a random intercept for each patient and a random slope for time were included. A *p* value of < 0.05 was considered significant.

## Results

### Patient characteristics and anthropometric findings at baseline

A total of 132 pediatric KTx patients (51 female and 81 male) were eligible for analysis (Table 1). Their mean age at baseline was 10.8 ± 4.4 years, and their mean weight and BMI *z*-scores amounted to 38.4 ± 18.9 kg and 0.19 ± 1.20, respectively. The percentage of overweight and obese patients amounted to 9% and 11%, respectively, which aligns to perceived trends in the German pediatric general population [8]. According to our data (Table 1), female adolescents show the highest percentage of overweight (9%) and obesity (26%). Interestingly, 19% of male adolescents were underweight, which rarely occurred in the other groups (4 and 5%, respectively). In the multivariable analysis, the BMI *z*-score was not associated with gender (*β* =  − 0.14, 95% CI − 0.22 to 0.02, *p* = 0.633), but with adolescence (≥ 12 years; *β* =  − 0.73, 95% CI − 1.24 to − 0.23, *p* = 0.006). Furthermore, a significant positive interaction of female gender and adolescent age on the BMI *z*-score was revealed (*β* = 1.20, 95% CI 0.38 to 2.02, *p* = 0.005) (Table 2). Combining these findings, female adolescents presented with higher mean BMI *z*-scores compared with male adolescents (difference: − 1.05, 95% CI − 1.86 to − 0.24, *p* = 0.004), while no differences were noted between the other groups.

**Table 1:** Baseline characteristics of 132 pediatric kidney transplant recipients

	Total cohort	< = 12 years		> 12 years	
		Male	Female	Male	Female
*n*	132	38	28	43	23
Age (years)	10.8 (4.4)	6.5 (2.9)	8.0 (3.1)	14.4 (1.7)	14.3 (1.5)
BMI (kg/m^2^)	19.0 (4.5)	16.9 (2.4)	17.9 (4.1)	19.4 (3.7)	23.2 (5.9)
BMI (*z*-score)	0.19 (1.20)	0.34 (1.01)	0.31 (1.11)	-0.32 (1.17)	0.73 (1.38)
Weight category (%)					
Underweight	12 (9)	2 (5)	1 (4)	8 (19)	1 (4)
Normal weight	94 (71)	27 (71)	22 (77)	31 (72)	14(61)
Overweight	12 (9)	6 (16)	2 (7)	2 (5)	2 (9)
Obese	14 (11)	3 (8)	3 (11)	2 (5)	6 (26)

Data are given as mean (SD) or *n* (%)

**Table 2:** Predictors of BMI *z*-score during COVID-19 pandemic in a cohort of 132 pediatric kidney transplant recipients

Predictors	Estimate	CI	*p*
(Intercept)	0.42	0.05–0.79	0.029
Age ≥ 12 years	− 0.73	− 1.24 to -0.23	**0.006**
Female gender	− 0.14	− 0.22 to 0.02	0.633
Age ≥ 12 years * female	1.20	0.38–2.01	**0.005**
Months since start	− 0.01	− 0.02 to 0.00	0.228
Age ≥ 12 years * months since start	0.02	0.00–0.03	**0.045**
Months since start * female	0.01	− 0.01 to 0.03	0.492
Months since start * age ≥ 12 years * female	− 0.01	− 0.03 to 0.02	0.596

### The course of BMI z-scores during the COVID-19 pandemic

**Fig. 1:**
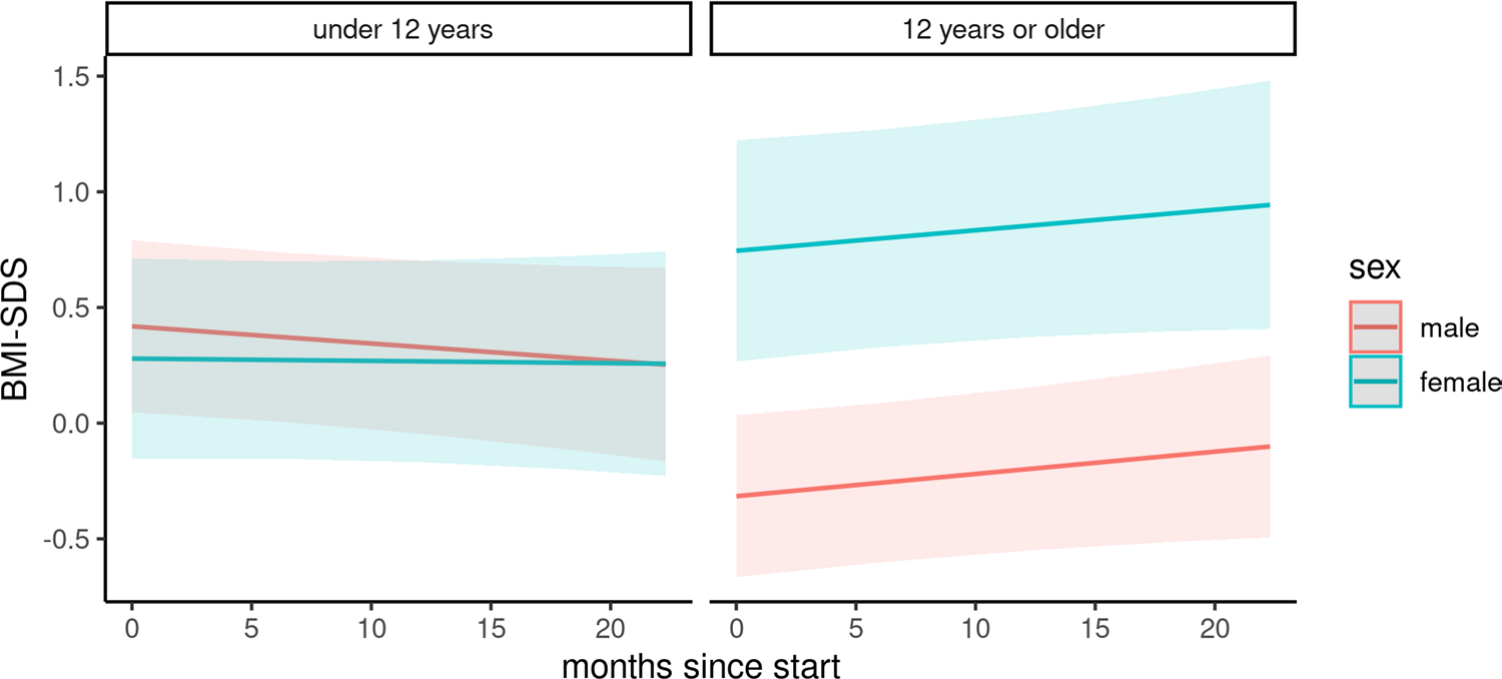
Course of BMI z-score

During the COVID-19 pandemic, no significant change in the BMI *z*-scores was observed in patients under 12 years of age, irrespective of gender (Fig. 1). In contrast, a significant increase in the mean BMI *z*-score was noted in both male and female adolescents (difference: male, 0.23, 95% CI 0.18 to 0.28; female 0.21, 95% CI 0.14 to 0.29, each *p* < 0.001). Consequently, when compared to the baseline, the percentage of obese female adolescents further increased from 26 to 33% and the percentage of underweight male adolescents decreased from 19 to 16%. However, this observation did not reach a level of statistical significance (each *p* > 0.05). The BMI *z*-score during the observation period was associated with adolescence as well as with a combination of adolescent age, female gender, and the duration of the pandemic (Table 2). In contrast, gender and duration of follow-up displayed no significant correlations per se (Table 1).

### Blood pressure

**Fig. 2:**
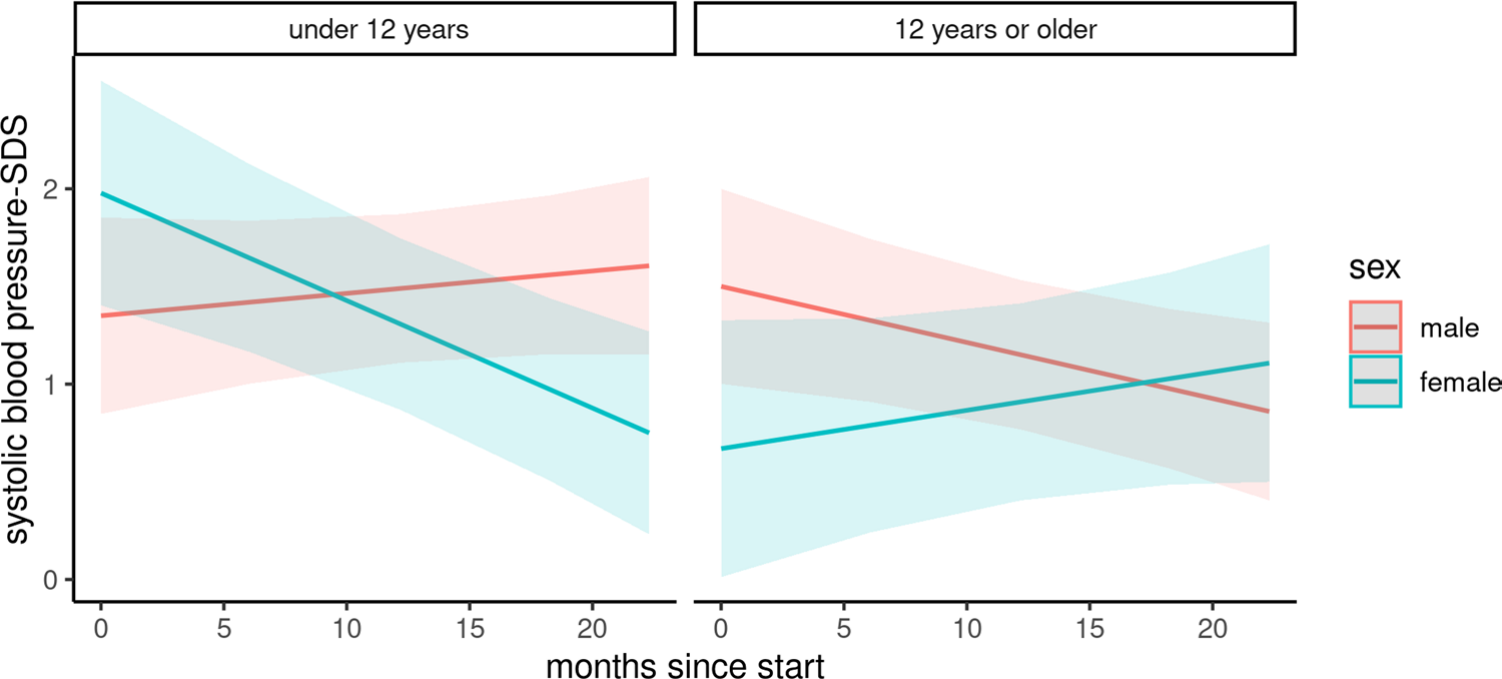
Course of systolic blood pressure z-score

**Fig. 3:**
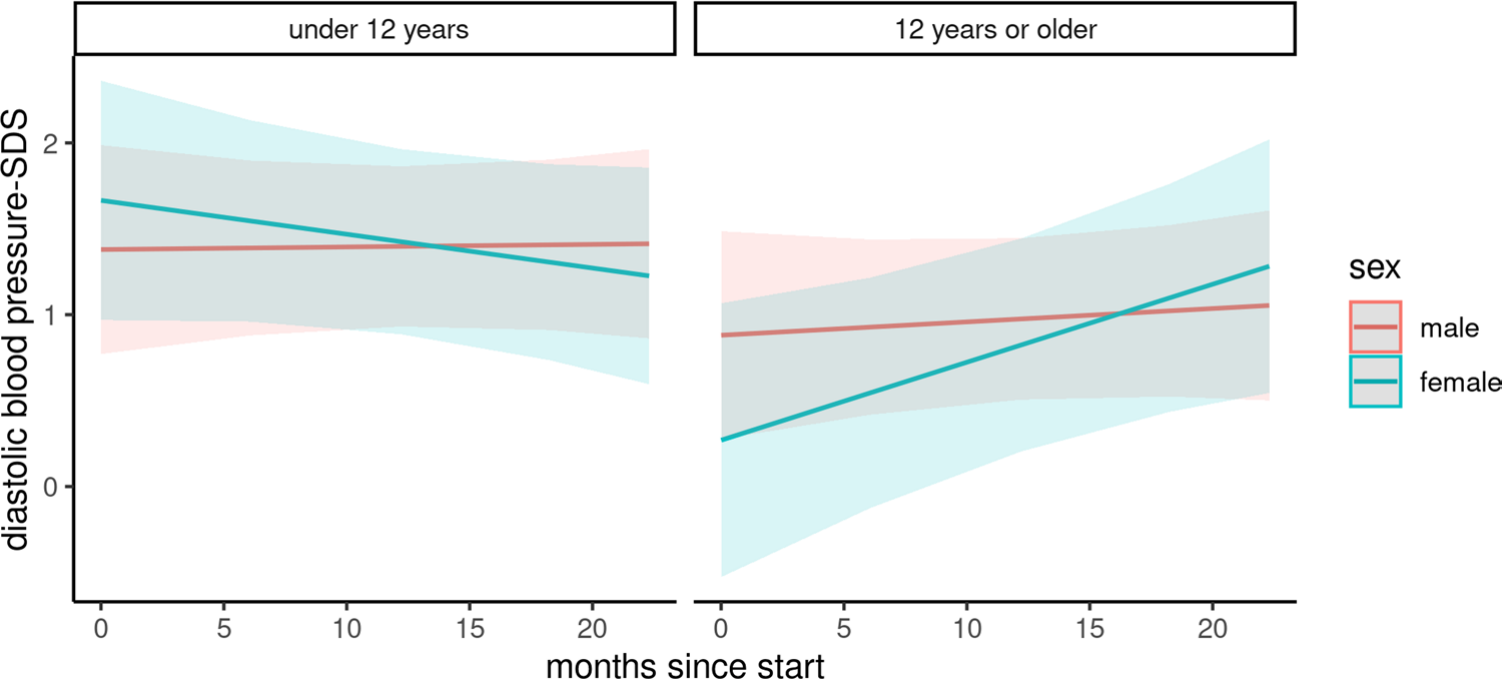
Course of diastolic blood pressure z-score

Changes in systolic and diastolic blood pressure were analyzed in a subcohort of 104 children (Figs. 2 and 3). The baseline characteristics of this subcohort are depicted in Table S1. During the COVID-19 pandemic, the systolic blood pressure *z*-score was negatively associated with the female gender and adolescent age and positively associated with the duration of the pandemic in combination with adolescent age and the female gender (Table 3). In summary, the mean systolic blood pressure significantly increased among boys under 12 years of age (difference: 0.28, 95% CI 0.25 to 0.30) and female adolescents (difference: 0.47, 95% CI 0.46 to 0.49). In contrast, systolic blood pressure decreased in females under 12 years of age (difference: − 1.32, 95% CI − 1.29 to − 1.35) and adolescent boys (difference: − 0.69, 95% CI − 0.67 to − 0.71). No significant changes were noted in diastolic blood pressure among the groups.

**Table 3:** Predictors of blood pressure *z*-score in a cohort of 106 pediatric kidney transplant recipients at baseline and during the COVID-19 pandemic

Predictors	Systolic blood pressure			Diastolic blood pressure		
	Estimate	CI	*p*	Estimate	CI	*p*
(Intercept)	1.35	0.85–1.85	< 0.001	1.37	0.78–1.98	< 0.001
Age ≥ 12 years	0.15	− 0.55 to 0.85	0.679	− 0.50	− 1.34 to 0.35	0.256
Female gender	0.63	− 0.13 to 1.38	0.110	0.29	− 0.63 to 1.20	0.546
Age ≥ 12 years * female	− 1.46	− 3.19 to 0.00	**0.012**	− 0.90	− 2.24 to 0.45	0.200
Months since start	0.01	− 0.01 to 0.04	0.387	0.10	− 0.03 to 0.03	0.924
Months since start * age ≥ 12 years	− 0.04	− 0.08 to − 0.00	**0.033**	0.00	− 0.04 to 0.05	0.776
Months since start * female	− 0.02	− 0.11 to − 0.03	**0.001**	− 0.07	− 0.07 to − 0.02	0.370
Months since start * age ≥ 12 years * female	0.11	0.06–0.17	** < 0.001**	0.06	− 0.01 to 0.13	0.094

Data were analyzed by linear mixed model

### Serum lipids

**Fig. 4:**
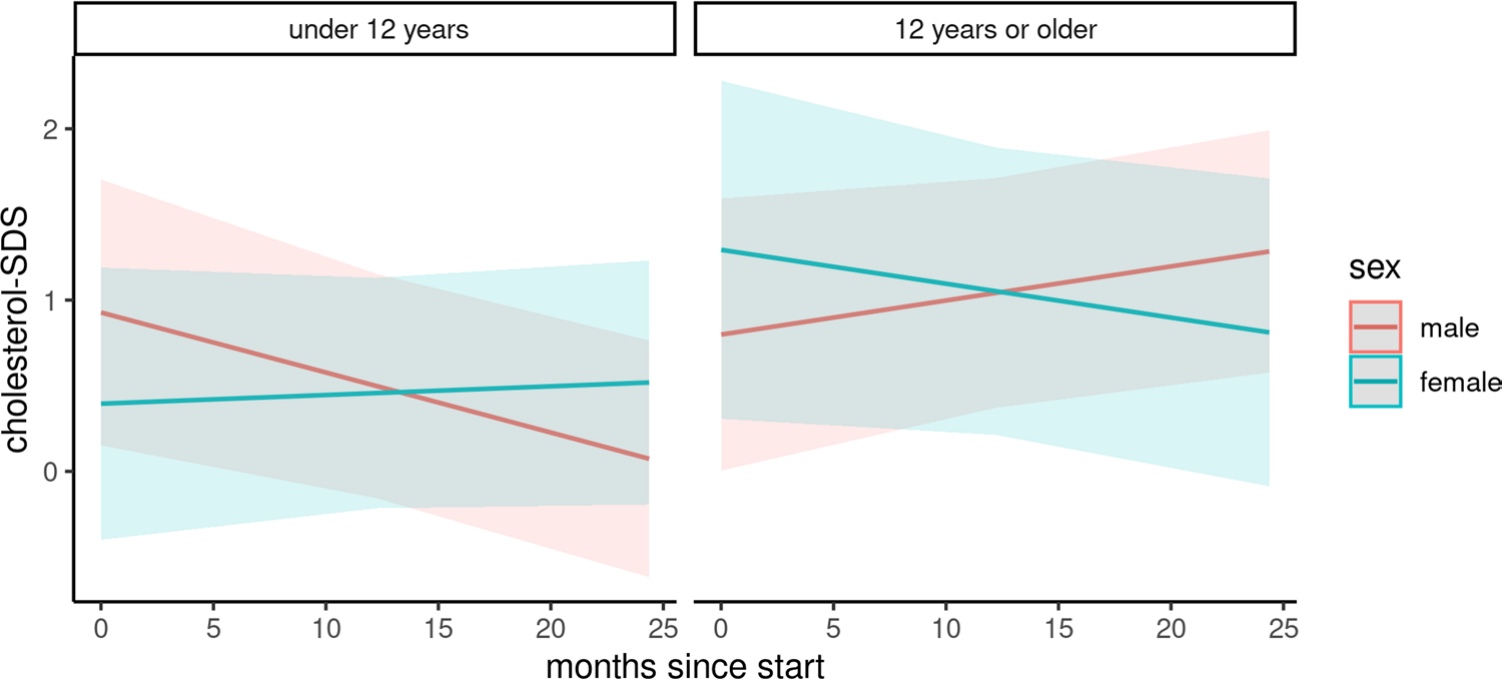
Course of cholesterol z-score

**Fig. 5:**
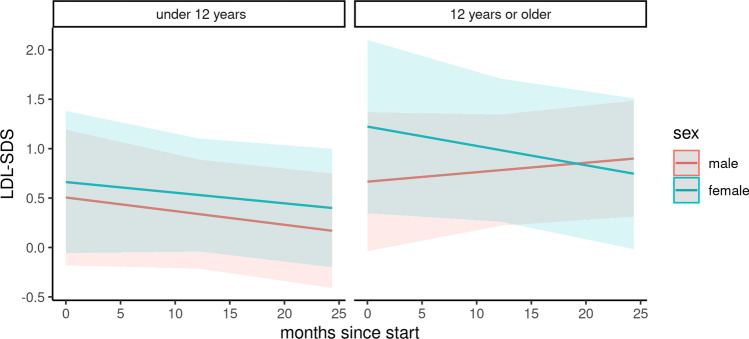
Course of LDL z-score

**Fig. 6:**
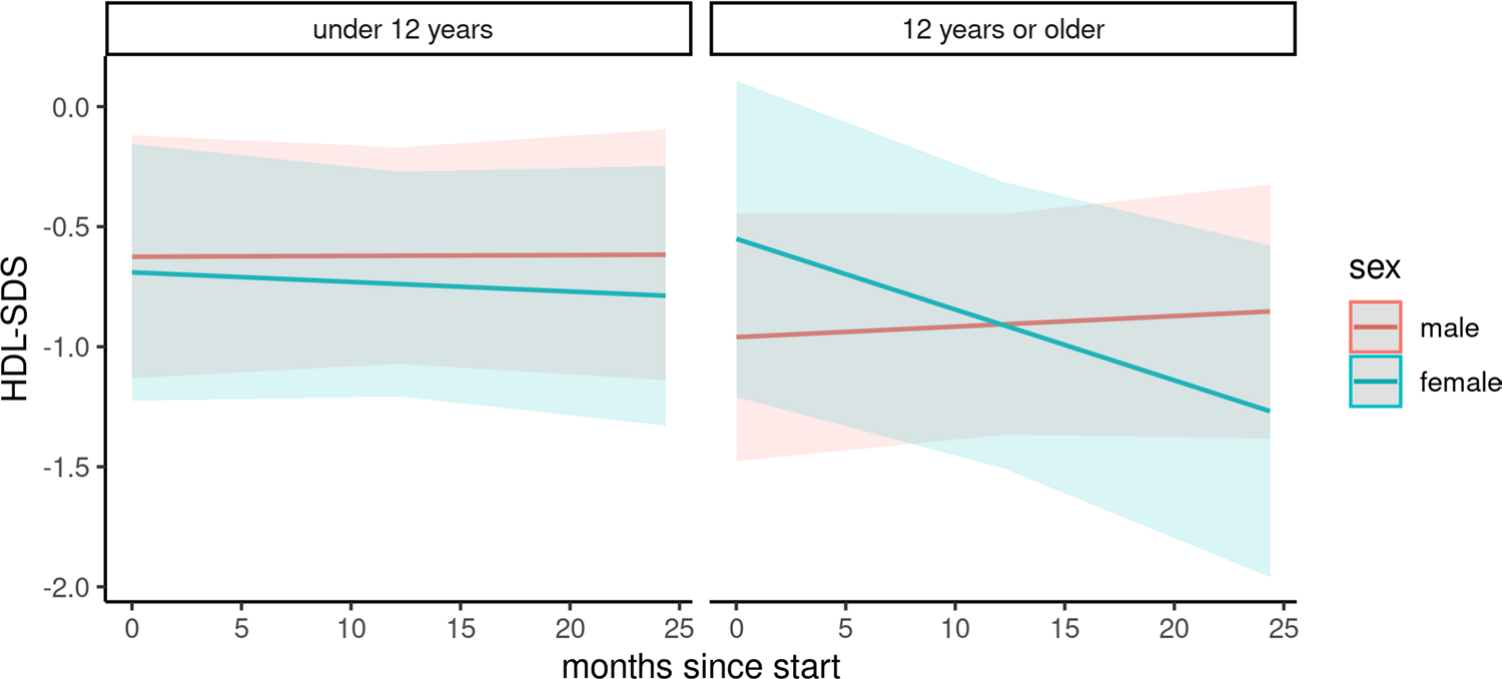
Course of HDL z-score

Changes in serum lipids were analyzed in a subcohort of 74 children (Figs. 4, 5, and 6). The baseline characteristics of this subcohort are depicted in Table S2. During the COVID-19 pandemic, the total cholesterol *z*-score was positively associated with female gender and adolescent age. It was further negatively associated with the duration of the pandemic, combined with adolescent age and female gender (Table 4). According to our results, the total cholesterol significantly increased in female patients under 12 years of age (difference: 0.12, 95% CI 0.04 to 0.21) and in male adolescents (difference: 0.48, 95% CI 0.39 to 0.57). The total cholesterol decreased in boys under 12 years of age (difference: − 0.84, 95% CI − 0.75 to − 93) and in female adolescents (difference − 0.47, 95% CI − 0.38 to − 0.57). No significant changes were found for low-density lipoprotein cholesterol and high-density lipoprotein cholesterol levels.

**Table 4:** Predictors of total cholesterol, high-density lipoprotein cholesterol, and low-density lipoprotein cholesterol *z*-scores in a cohort of 74 pediatric kidney transplant recipients at baseline and during the COVID-19 pandemic

Predictors	Total cholesterol			High-density lipoprotein cholesterol			Low-density lipoprotein cholesterol		
	Estimate	CI	*p*	Estimate	CI	*p*	Estimate	CI	*p*
(Intercept)	0.93	0.16–1.69	0.021	0.51	− 0.16 to 1.18	0.154	− 0.*63*	− 1.12 to − 0.127	0.018
Age ≥ 12 years	− 0.13	− 1.22 to 0.97	0.821	1.60	− 0.81 to 1.13	0.750	− 0.33	− 1.05 to 0.38	0.368
Female gender	− 0.53	− 1.62 to 0.56	0.350	0.16	− 0.84 to 1.13	0.759	− 0.07	− 0.79 to 0.66	0.863
Age ≥ 12 years * female	1.03	− 0.64 to 2.69	0.236	0.40	− 1.07 to 1.89	0.603	0.47	− 0.63 to 1.57	0.407
Months since start	− 0.04	− 0.06 to − 0.00	0.014	− 0.01	− 0.04 to 0.01	0.298	0.00	− 0.02 to 0.02	0.973
Months since start * age ≥ 12 years	0.05	0.02–0.09	0.008	0.02	− 0.01 to 0.06	0.218	0.0*0*	− 0.02 to 0.03	0.784
Months since start * female	0.04	0.00–0.08	0.048	0.00	− 0.03 to 0.04	0.875	− 0.00	− 0.03 to 0.02	0.775
Months since start * age ≥ 12 years * female	− 0.0*8*	− 0.14 to 0.02	0.011	− 0.03	− 0.09 to 0.02	0.264	− 0.03	− 0.07 to 0.01	0.197

## Discussion

In this retrospective cohort study, we specifically investigated the period of the COVID-19 pandemic with its associated lockdowns. We found a significant increase in BMI *z*-scores in adolescents over 12 years of age, regardless of gender. However, we did not find significant changes in younger children within our study sample. Furthermore, we could not identify a uniform, synchronous pattern regarding changes in the *z*-scores of blood pressure and cholesterol within our population.

The significant increase in BMI *z*-scores in adolescents after KTx surprisingly opposes a large German study conducted with a healthy pediatric population [8]. The researchers identified significant weight gains during the COVID-19 pandemic in age groups under 12 years of age, but not in adolescents between 12 and 18 years of age. However, a Palestinian study reported significant weight gains in adolescents, thus underlining our results. The researchers associated this trend with the increased consumption of unhealthy food, such as fast food [15]. Although we did not evaluate the diets of our patients, we also suspect changes in nutrition during the COVID-19 pandemic in the German context. We assume that the closing of schools and restrictions on sporting activities, such as closing of indoor clubs, changed the level of physical activity, diet, and eating habits. This likely affects adolescents more than younger children. Furthermore, personal contact restrictions leading to increased distance from friends and family members could potentially weigh heavier on adolescents [16]. Another relevant factor could be high levels of psychological stress, resulting in altered eating behaviors. Several researchers emphasize that the COVID-19 pandemic raised anxieties and stress levels among both parents and their children [1, 17, 18]. It could be speculated that this especially affected adolescent girls, and that they thus display more considerable changes in eating habits and physical movement than other age groups [19, 20]. However, we were puzzled by the high percentage of obese adolescent girls in our study. The causes for this distribution remain unclear and require further investigation. To speculate, the COVID-19 pandemic and its associated physical and psychological strains likely worsened existing problems surrounding body awareness and self-esteem, which seemingly influence girls more than boys.

Psychological stress and concerns about COVID-19 could have affected adolescents after KTx and their parents profoundly because at the beginning of the pandemic, the meaning and risks associated with a COVID-19 infection in this cohort were unknown. Thus, this demographic could have voluntarily enhanced the official lockdown measures to further protect themselves from a COVID-19 infection. Those considerations and the resulting psychological stress are less relevant for younger children. Those children are used to accepting their personal situations, and psychological effects often only concern their parents. This might explain why psychological effects are less significant in the younger KTx cohort (< 12 years).

Compared to the population average, patients after KTx have a dramatically higher risk of cardiovascular disease, which is the main cause of death with functioning grafts worldwide [21]. Typical cardiovascular risk factors, like hypertension, diabetes mellitus, and dyslipidemia, summarized as metabolic syndrome, are prevalent in patients with chronic kidney disease [22]. This risk prevails after KTx. Typical post-transplant medication-included adverse effects are hypertension, post-transplantation diabetes, and hyperlipidemia, which are all well-recognized cardiovascular risk factors [23]. Obesity showed an increased risk of graft failure and mortality in patients who were overweight after KTx [24].

Infants after KTx experience greater nutritional problems during their time of chronic kidney failure. Most require a special protein, potassium, and phosphate-reduced diet, which leads to feeding difficulties and often manifests in later eating disorders. According to an Italian study, the energy intake in patients with chronic kidney failure is 10% lower, and their protein intake is 33% lower than in healthy children [25]. These preexisting issues might add to the weight gain in addition to inactivity and other psychological factors.

The most urgent question for pediatricians is how to support children and adolescents with significant weight gain. A large KTx 360° cohort study from Hannover revealed an association between increasing BMI and decreasing graft function [26]. Thus, weight gain poses a high risk for pediatric patients after KTx.

We recommend implementing a healthy weight loss program for all overweight pediatric patients after KTx. The program should be supervised by a dietician and include individual nutrition plans. Additionally, patients should be supported by members of the department of sports medicine and start a physical activity program. A smartwatch could be added to the program in order to help patients count their steps and to incorporate more movement in their daily routines. Finally, it should be our aim to identify high-risk patients who could benefit from intensified guidance and education at an early stage of being overweight to reduce their risk of progression to associated comorbidities.

The limitations of this study include the lack of comprehensive documentation regarding medication and the lack of a dietary diary. Therefore, we could only speculate as to what caused the significant weight gain in adolescents during the COVID-19 pandemic. Another limitation lies in our retrospective study design. Since the focus of our analysis was the BMI scores, we included blood pressure and cholesterol as the nearest and most commonly measured variables. Unfortunately, we could not obtain blood pressure measurements and cholesterol levels for all patients. This was due to the retrospective study design, differing schedules for blood tests across the participating pediatric nephrology centers, and restrictions caused by the COVID-19 pandemic on regular patient visits. Further research could expand the data to other parameters, such as triglyceride levels or serum glucose levels, which were not available to us.

The strength of our study is that to our knowledge, this was the first attempt to map the impact of the COVID-19 pandemic on body mass index in children and adolescents after KTx. In relation to pediatric kidney transplant cohorts, we managed to describe a large German cohort that was periodically assessed. We thereby identified adolescent girls as a particularly vulnerable population in kidney-transplanted children. Our observations concerning weight gain provide a relevant foundation for long-term follow-up studies. This is vital because even without the COVID-19 pandemic, children were already known to have an increased risk of obesity after solid organ transplantation. This is particularly relevant for pediatric KTx patients, for whom the adjusted cumulative prednisone use was shown to be associated with an increased risk of obesity [6].

In conclusion, adolescents after KTx showed a significant increase in BMI *z*-score during the COVID-19 pandemic, which was associated with an increase in systolic blood pressure in female adolescents. This further increases the risks for cardiovascular diseases in this specific patient population.

## Supplementary Information

Below is the link to the electronic supplementary material.

Graphical Abstract (PPTX 95 kb)

Supplementary file2 (DOCX 17 kb)

Supplementary file3 (DOCX 16 kb)

## Data Availability

The datasets generated during and/or analysed during the current study are available from the corresponding author on reasonable request.

## Abbreviations

COVID-19: Coronavirus SARS-CoV-2-disease
BMI: Body mass index
KTx: Kidney transplantation
CsA: Cyclosporine A
MMF: Mycophenolate mofetil
EVR: Everolimus
TAC: Tacrolimus
