# Supplementary material for: Impact of the COVID-19 pandemic on body mass index in children and adolescents after kidney transplantation
Source: Pediatr Nephrol. 2023 Mar 2;38(8):2801–8. doi: 10.1007/s00467-023-05902-4 (PMC9979889; doi:10.1007/s00467-023-05902-4)
Supplement: Supplementary file 1 — Graphical Abstract (PPTX 95 kb) [file 467_2023_5902_MOESM1_ESM.pptx]

## Slide 1
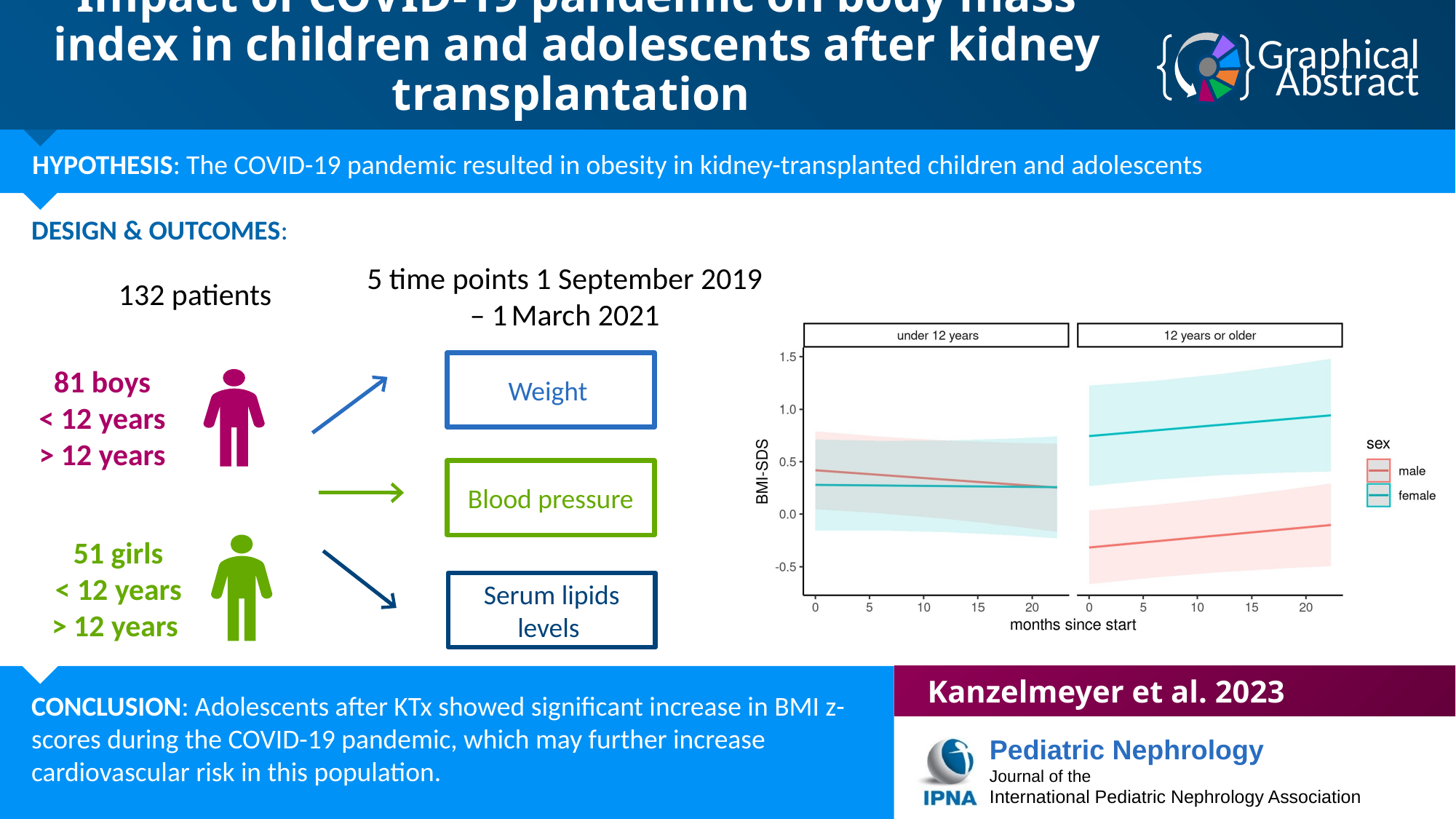

Impact of COVID-19 pandemic on body mass index in children and adolescents after kidney transplantation
HYPOTHESIS: The COVID-19 pandemic resulted in obesity in kidney-transplanted children and adolescents
DESIGN & OUTCOMES:
5 time points 1 September 2019 – 1 March 2021
132 patients
Weight
81 boys
< 12 years
> 12 years
Blood pressure
51 girls
< 12 years
> 12 years
Serum lipids levels
Kanzelmeyer et al. 2023
CONCLUSION: Adolescents after KTx showed significant increase in BMI z-scores during the COVID-19 pandemic, which may further increase cardiovascular risk in this population.
