# Supplementary material for: Impact of the COVID-19 pandemic on body mass index in children and adolescents after kidney transplantation
Source: Pediatr Nephrol. 2023 Mar 2;38(8):2801–8. doi: 10.1007/s00467-023-05902-4 (PMC9979889; doi:10.1007/s00467-023-05902-4)
Supplement: Supplementary file 2 — Supplementary file2 (DOCX 17 kb) [file 467_2023_5902_MOESM2_ESM.docx]

**Table S1: Baseline characteristics of 104 pediatric kidney transplant recipients included in the analysis of blood pressure**

|  | Total cohort | <= 12 years | | > 12 years | |
| --- | --- | --- | --- | --- | --- |
|  |  | male | female | male | female |
| n | 104 | 31 | 24 | 31 | 18 |
| Age (years) | 10.2 (4.4) 0.8 (4.4) | 5.9 (2.6) | 7.9 (3.2) | 14.0 (1.5) | 14.2 (1.3) |
| BMI (kg/m^2^) | 18.7 (4.2) | 16.6 (1.7) | 17.6 (3.8) | 19.3 (3.9) | 22.8 (5.2) |
| BMI (z-score) | 0.19 (1.15) | 0.32 (0.98) | 0.24 (1.05) | -0.25 (1.05) | 0.64 (1.34) |
| Weight category (%) |  |  |  |  |  |
| Underweight | 7 (7) | 1 (3) | 1 (4) | 4 (13) | 1 (6) |
| Normal weight | 79 (76) | 23 (74) | 20 (83) | 25 (81) | 11 (61) |
| Overweight | 8 (8) | 5 (16) | 1 (4) | 0 (0) | 2 (11) |
| Obese | 10 (10) | 2 (7) | 2 (8) | 2 (7) | 4 (22) |
| Systolic blood pressure (z-score) | 1.32 (1.61) | 1.19 (1.46) | 1.78 (1.45) | 1.52 (1.20) | 0.57 (2.36) |
| Diastolic blood pressure (z-score) | 1.04 (1.89) | 1.31 (1.88) | 1.53 (2.01) | 0.99 (1.31) | 0.01 (2.30) |

Data are given as mean (SD) or n (%).
