# Supplementary material for: Impact of the COVID-19 pandemic on body mass index in children and adolescents after kidney transplantation
Source: Pediatr Nephrol. 2023 Mar 2;38(8):2801–8. doi: 10.1007/s00467-023-05902-4 (PMC9979889; doi:10.1007/s00467-023-05902-4)
Supplement: Supplementary file 3 — Supplementary file3 (DOCX 16 kb) [file 467_2023_5902_MOESM3_ESM.docx]

**Table S2: Baseline characteristics of 74 pediatric kidney transplant recipients included in the analysis of lipids**

|  | Total cohort | <= 12 years | | > 12 years | |
| --- | --- | --- | --- | --- | --- |
|  |  | male | female | male | female |
| n | 74 | 22 | 17 | 20 | 15 |
| Age (years) | 10.4 (4.2) 0.8 (4.4) | 6.4 (3.5) | 8.3 (2.8) | 13.9 (1.1) | 14.0 (1.2) |
| BMI (kg/m^2^) | 18.9 (4.2) | 16.5 (1.6) | 18.2 (4.7) | 19.1 (2.5) | 23.2 (5.1) |
| BMI (z-score) | 0.21 (1.09) | 0.12 (0.82) | 0.31 (1.13) | -0.23 (0.97) | 0.79 (1.36) |
| Weight category (%) |  |  |  |  |  |
| Underweight | 4 (5) | 0 (0) | 0 (0) | 2 (10) | 2 (13) |
| Normal weight | 59 (80) | 20 (91) | 14 (82) | 18 (90) | 7 (47) |
| Overweight | 5 (7) | 2 (9) | 1 (6) | 0 (0) | 2 (13) |
| Obese | 6 (8) | 0 (0) | 2 (12) | 0 (0) | 4 (27) |
| Total cholesterol (z-score) | 0.85 (1.63) | 0.53 (1.53) | 0.72 (1.34) | 0.88 (1.45) | 1.47 (2.23) |
| LDL-cholesterol (z-score) | 0.74 (1.48) | 0.24 (1.31) | 0.86 (1.12) | 0.80 (1.19) | 1.26 (2.21) |
| HDL-cholesterol (z-score) | -0.75 (1.20) | -0.60 (1.07) | -0.75 (1.39) | -1.02 (1.46) | -0.61 (0.79) |

Data are given as mean (SD) or n (%).
